# Mitochondrial content and intrinsic coupling‐related differences shape bioenergetic output and redox responses across striated muscles in rats

**DOI:** 10.1113/EP094334

**Published:** 2026-11-03

**Authors:** Alex Chan, Jules Devaux, Claudia Wen, Anthony Phillips, Anthony Hickey, Toan Pham

**Affiliations:** ^1^ Auckland Bioengineering Institute The University of Auckland Auckland New Zealand; ^2^ School of Biological Sciences Faculty of Science The University of Auckland Auckland New Zealand

**Keywords:** antioxidant, diaphragm, heart, mitochondria, ROS, skeletal

## Abstract

Striated muscles differ markedly in energetic demand, but whether mitochondrial respiration and reactive oxygen species emission differ intrinsically across muscle types remains unclear. This study compared mitochondrial respiration, hydrogen peroxide (H_2_O_2_) emission and antioxidant enzyme expression in heart, diaphragm and hindlimb skeletal muscle from healthy rats. Mitochondrial O_2_ flux and H_2_O_2_ emission were measured simultaneously using high‐resolution respirometry coupled with fluorometry in tissue homogenates subjected to substrate‐uncoupler‐inhibitor titration protocols. Mitochondrial content was estimated by citrate synthase activity, and antioxidant enzyme expression was measured by immunoblotting. Heart tissue displayed the highest mitochondrial respiration and H_2_O_2_ emission when expressed per tissue mass, followed by diaphragm and skeletal muscle. However, normalisation by citrate synthase activity eliminated differences in respiratory flux between muscles, indicating that variations in tissue‐level respiration were primarily driven by mitochondrial content. Net H_2_O_2_ emission was greatest during leak respiration and decreased during oxidative phosphorylation and uncoupled states in all muscle types. Cardiac mitochondria exhibited a higher H_2_O_2_/O_2_ ratio during leak respiration and higher coupling‐related ratios than those of diaphragm and skeletal muscle. In association, cardiac muscle showed greater expression of the mitochondrial antioxidant enzymes superoxide dismutase 2 and catalase in a separate cohort. These findings indicate that absolute oxidative metabolism across striated muscles is largely determined by mitochondrial content, whereas respiratory capacity per mitochondrial unit was conserved. Cardiac muscle exhibits elevated leak‐associated reactive oxygen species emission but compensates by enhancing antioxidant defences, highlighting coordination between bioenergetic demand and redox regulation in continuously active muscles.

## Abstract (highlights)

- **What is the central question of this study?**
  How do mitochondrial respiration and redox response differ across various striated muscles?
- **What is the main finding and its importance?**
  By combining high‐resolution respirometry, fluorimetry and immunoblotting, this study shows that oxidative metabolism in striated muscles is mainly driven by mitochondrial density, with similar intrinsic respiratory capacity per mitochondrion. Cardiac tissue produces more leak‐related reactive oxygen species and, in a separate cohort, exhibits a stronger antioxidant defence capacity than diaphragm and limb skeletal muscle tissues, indicating tight regulation between energy demand and redox control in active muscle tissues.

## 1 INTRODUCTION

Striated muscles rely on mitochondrial oxidative phosphorylation to support ATP production for muscle contraction. Mitochondria are also a major intracellular source of reactive oxygen species (ROS) during aerobic respiration. A small fraction of oxygen is converted to superoxide during aerobic respiration, and physiological levels of oxidative stress can be balanced by cellular antioxidant defence systems, including superoxide dismutase and catalase (Galasso et al., 2021; Miao & St Clair, 2009; Murphy, 2009). Disruption of this redox balance drives oxidative damage and contributes to diverse pathologies, including metabolic, neurodegenerative and cardiovascular diseases (Houldsworth, 2024; Panth et al., 2016; Pham et al., 2014). Understanding how mitochondrial ROS emission and antioxidant defences vary across muscle types is therefore central to defining tissue‐specific redox vulnerability.

Striated muscles differ significantly in metabolic demand and mitochondrial density (Fernández‐Vizarra et al., 2011; Park et al., 2014). Cardiac muscle, operating continuously, exhibits the highest oxidative workload; skeletal muscle in the limbs adjusts energy use with locomotor activity; and the diaphragm occupies an intermediate role, combining rhythmic involuntary activity with voluntary control (Fogarty & Sieck, 2019; Wang et al., 2010). Previous work has reported higher mitochondrial respiration and ROS emission in cardiac tissue relative to skeletal muscle (Park et al., 2014; Li Puma *et al.*, 2020; Saqib et al., 2026). Reports on diaphragm mitochondrial function are inconsistent, including lower (Garcia‐Cazarin et al., 2011) or higher O_2_ flux (Hahn et al., 2019) compared to limb skeletal muscles, and few studies have normalised respiratory flux to mitochondrial content (Hahn et al., 2019), limiting mechanistic interpretation. Importantly, direct comparisons of mitochondrial redox responses and antioxidant defence across heart, diaphragm and limb muscle tissues remain elusive.

To address this gap, we directly compared mitochondrial respiration, H_2_O_2_ emission and antioxidant enzyme expression in heart, diaphragm and hindlimb skeletal muscle from healthy rats. Using high‐resolution respirometry and fluorometry, we tested the hypothesis that cardiac mitochondria exhibit greater respiratory output and antioxidant defence than those in other striated muscles. These data provide foundational insight into muscle‐specific mitochondrial redox phenotypes and establish a comparative framework for future studies of disease and therapeutic targeting.

## 2 METHODS

### 2.1 Ethical approval

All experiments were conducted in accordance with protocols approved by the University of Auckland Animal Ethics Committee (AEC22653) and conformed to institutional guidelines for animal experimentation.

### 2.2 Animals

Male Wistar rats (400–500 g; *n* = 11) were purchased from the animal facility (Vernon Jansen Unit) at the University of Auckland. They were housed in pairs in a 12/12 h light–dark cycle at 21°C with chow and tap water available ad libitum. Six animals were used for respirometry and citrate synthase analyses, and five for immunoblotting.

### 2.3 Sample preparation

Animals were anaesthetised with isoflurane (5% in oxygen gas) and euthanised by cervical dislocation. The left ventricular myocardium, diaphragm and hindlimb skeletal muscle (gracilis) were rapidly excised, immediately arrested in ice‐cold saline, and divided into two aliquots. The first set of fresh samples (20 mg) was stored in histidine–tryptophan–ketoglutarate buffer (Custodiol, Köhler Chemie, Alsbach‐Hähnlein, Germany) on ice prior to respirometry measurements. The other set of samples (100 mg) was rapidly frozen at −80°C for subsequent enzyme activity analysis. The tissues from five separate animals were immediately frozen at −80°C for immunoblotting.

Fresh tissues were quickly blotted on filter paper, weighed and transferred to 500 µL ice‐cold MiRO5 buffer (in mM: 110 sucrose, 60 K‐lactobionate, 20 HEPES, 20 taurine, 10 KH_2_PO_4_, 3 MgCl_2_, 0.5 EGTA, and 1 mg/mL fraction V BSA, pH 7.1 at 30°C). Samples were carefully homogenised for ∼10 s using a tissue homogeniser (Omni International, Kennesaw, GA, USA) and kept on ice before respirometry assays. All experiments were performed at 37°C.

### 2.4 Mitochondrial respiration and hydrogen peroxide emission

Three high‐resolution fluo‐respirometers (Oroboros Instruments, Innsbruck, Austria) were used to measure mitochondrial function. Exactly 2 mg of homogenate was added to each measurement chamber and allowed to reach equilibrium. A substrate–uncoupler–inhibitor titration protocol was used to measure mitochondrial respiration of 2 mg. Leak respiration with complex I substrates was initiated with malate (2 mM), glutamate (10 mM) and pyruvate (5 mM), followed by succinate (10 mM). Oxidative phosphorylation was stimulated with ADP (2.5 mM), uncoupled capacity was measured by stepwise carbonyl cyanide *m*‐chlorophenyl hydrazone titration (0.5 µM steps), and residual oxygen consumption was determined with antimycin A (1 µM). Cytochrome *c* oxidase (CCO) or Complex IV activity was assessed using ascorbate (2 mM) and *N*,*N*,*N*′,*N*′‐tetramethyl‐*p*‐phenylenediamine dihydrochloride (0.5 mM), followed by azide (100 mM) inhibition.

Net H_2_O_2_ emission rate was measured alongside respiration using Amplex UltraRed (AUR, 25 µM), horseradish peroxidase (10 U/mL) and superoxide dismutase (5 U/mL), as previously described (Pham et al., 2026).

### 2.5 Citrate synthase activity and immunoblotting analysis

Citrate synthase activity was measured from frozen samples (∼30 mg) as previously described (Pham et al., 2020), using a BioTek microplate reader (BioTek® Instruments, Winooski, VT, USA), and normalised to total soluble protein content using a BCA‐protein kit (Thermo Fisher Scientific, Waltham, MA, USA).

Immunoblotting analysis for catalase and superoxide dismutase 2 (SOD2) was performed as previously described (Fang et al., 2026), using frozen samples (∼40 mg; *n* = 5). Antibodies were purchased from Abcam (Waltham, MA, USA; ab16731 and ab13498 for catalase and SOD2, respectively) and diluted to 1:1000. Total protein staining was performed with amido black rather than a reference target to enable comparisons between tissue types.

### 2.6 Data and statistical analyses

Fluorimetry‐coupled respirometry data were recorded using DatLab 7.1 software (Oroboros Instruments). All respiration rates were corrected for residual O_2_ consumption rate after addition of antimycin A. CCO respiration was corrected with complex IV inhibition. To account for non‐mitochondrial H_2_O_2_ emission, all H_2_O_2_ measurements were corrected with the background H_2_O_2_ signal measured before the addition of homogenates. All data were normalised to tissue wet mass and citrate synthase activity. Statistical analyses and plotting were performed using Prism 11.0 (GraphPad Software, Boston, MA, USA). All data are presented as means ± SD unless stated otherwise. One‐way or two‐way repeated‐measures ANOVA with Tukey *post hoc* analyses were used to test differences between muscle groups and/or mitochondrial states, with *P* < 0.05 considered significant.

## 3 RESULTS

### 3.1 Mitochondrial respiration

Figure 1a represents typical raw data traces from the respiratory and fluorometry measurements of heart mitochondria. When comparing tissue‐mass specific O_2_ fluxes, we found O_2_ flux was significantly highest in the heart, followed by the diaphragm, and lowest in skeletal muscle in all respiratory states (*P* < 0.0138) (Figure 1b). However, when normalised to citrate synthase activities, there were no significant differences between muscle groups (Figure 1c), indicating that the differences in mitochondrial O_2_ flux per tissue mass resulted mostly from quantitative differences in mitochondrial content.

**FIGURE 1:**
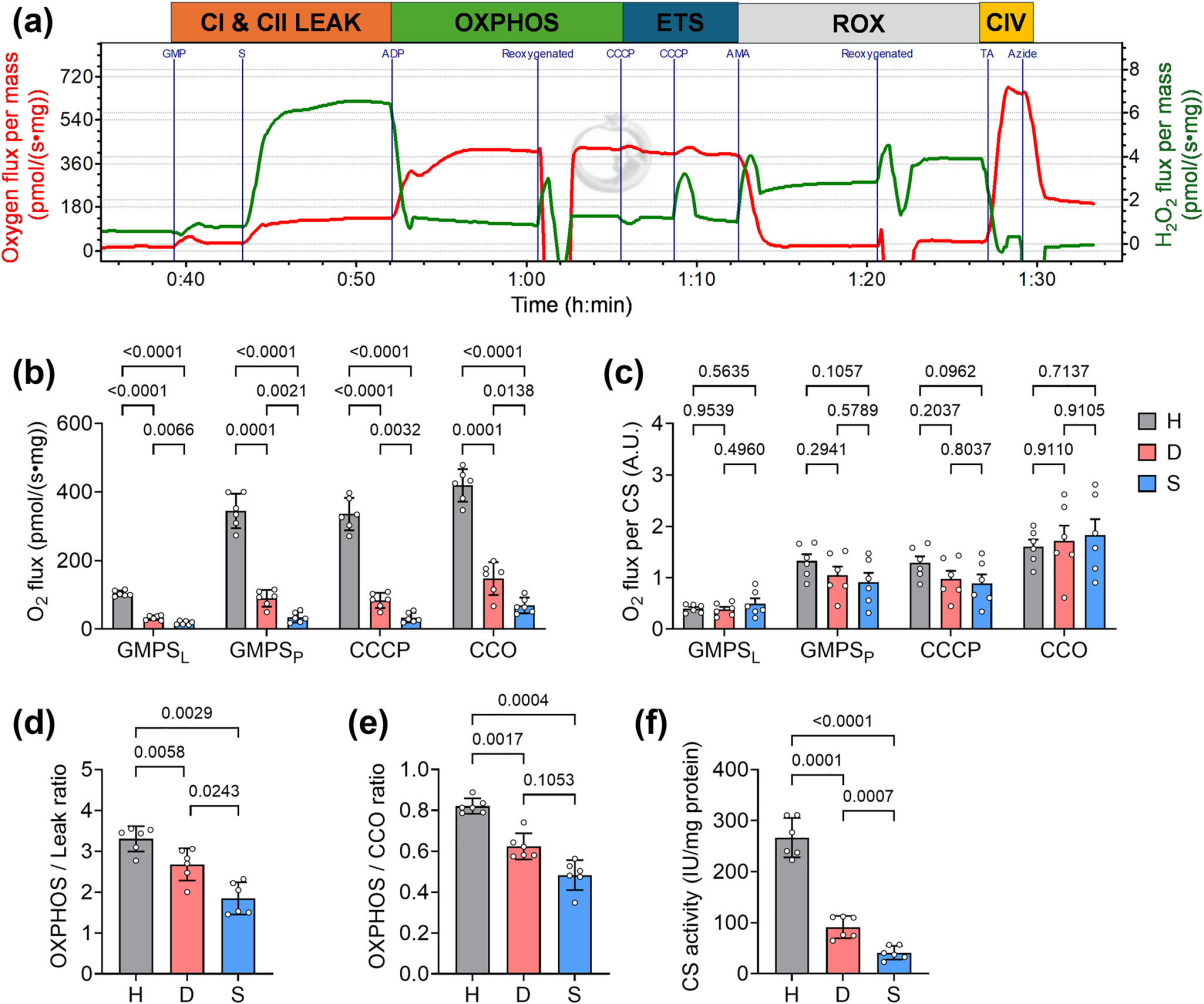
Mitochondrial respiration and efficiency in striated muscle types. (a) Typical experimental records from heart homogenate, with mitochondrial O_2_ flux (red line) and the corresponding H_2_O_2_ flux (green line), normalised by tissue wet mass. (b, c) Mitochondrial O_2_ flux normalised to tissue wet mass (b) and citrate synthase activity (c). (d) OXPHOS/leak ratio was calculated as a fraction of O_2_ flux in OXPHOS to leak respiratory with complex I and complex II substrates. (e) OXPHOS/CCO ratio was calculated as a fraction of O_2_ flux in OXPHOS to complex IV activity stimulated with *N*,*N*,*N*′,*N*′‐tetramethyl‐*p*‐phenylenediamine dihydrochloride and ascorbate. (f) Enzymatic activities of citrate synthase (CS) as an index of mitochondrial content, IU (µmol/min). D, diaphragm; H, heart; S, skeletal muscle. Two‐way ANOVA was used for (b, c); one‐way ANOVA was used for (d, e, f); both analyses were with Tukey *post hoc* analysis. Values are presented as means ± SD, *n* = 6. ADP, adenosine diphosphate; AMA, antimycin A; CCCP, carbonyl cyanide *m*‐chlorophenyl hydrazone; CCO, cytochrome *c* oxidase; ETS, electron transport system; GMPSL, glutamate, malate, pyruvate, succinate – leak respiration; GMPSP, glutamate, malate, pyruvate, succinate – OXPHOS respiration; ROX, residual oxygen consumption rate; TA, *N*,*N*,*N*',*N*’‐tetramethyl‐*p*‐phenylenediamine dihydrochloride and ascorbate.

The oxidative phosphorylation (OXPHOS)/leak ratio is a measure of coupling efficiency, defined as the proportion of mitochondrial O_2_ flux used to synthesise ATP, calculated by dividing the leak respiration rate by OXPHOS respiration rate when O_2_ flux mainly compensates for proton flow not utilised for ATP synthesis. This ratio was highest in heart mitochondria, followed by the diaphragm, and lowest in skeletal muscle (*P* = 0.0243) (Figure 1d). In our protocols, a combination of *N*,*N*,*N*',*N*’‐tetramethyl‐*p*‐phenylenediamine dihydrochloride and ascorbate was used to obtain maximal O_2_ flux through complex IV (cytochrome *c* oxidase). The ratio of O_2_ flux in OXPHOS state to complex IV activity is a key indicator of mitochondrial reserve capacity over the overall OXPHOS capacity. The heart mitochondria showed the highest OXPHOS/CCO ratio among tissues (*P* = 0.0017), indicating a more efficient O_2_ flux to drive ATP synthesis (Figure 1e). OXPHOS/CCO ratio did not differ between diaphragm and skeletal muscle (*P* = 0.1053). CS activities were highest in the heart, ∼3‐fold greater than in the diaphragm tissue and 5‐fold greater than in skeletal muscle (*P* < 0.0001) (Figure 1f).

### 3.2 Mitochondrial H_2_O_2_ emission and endogenous antioxidant enzymes

Net mitochondrial H_2_O_2_ levels were considerably higher in the leak states than in the OXPHOS and uncoupled states, regardless of muscle type (Figure 2a). When normalised to tissue mass, mitochondria from the heart produced the highest rate of H_2_O_2_, ∼6‐fold greater than that of the diaphragm and ∼10‐fold greater than that of skeletal muscle in all respiratory states. However, when normalised to CS activity, heart mitochondria showed higher H_2_O_2_ emission rates only in the leak respiratory state compared with other muscle types, with no difference between the OXPHOS and uncoupled states (Figure 2b). Heart mitochondria exhibited the highest H_2_O_2_/O_2_ ratio, followed by the diaphragm, and the lowest in muscle, only in the leak state, with no difference in OXPHOS or uncoupled state, indicating greater H_2_O_2_ leakage than O_2_ consumption in the leak state (Figure 2c).

**FIGURE 2:**
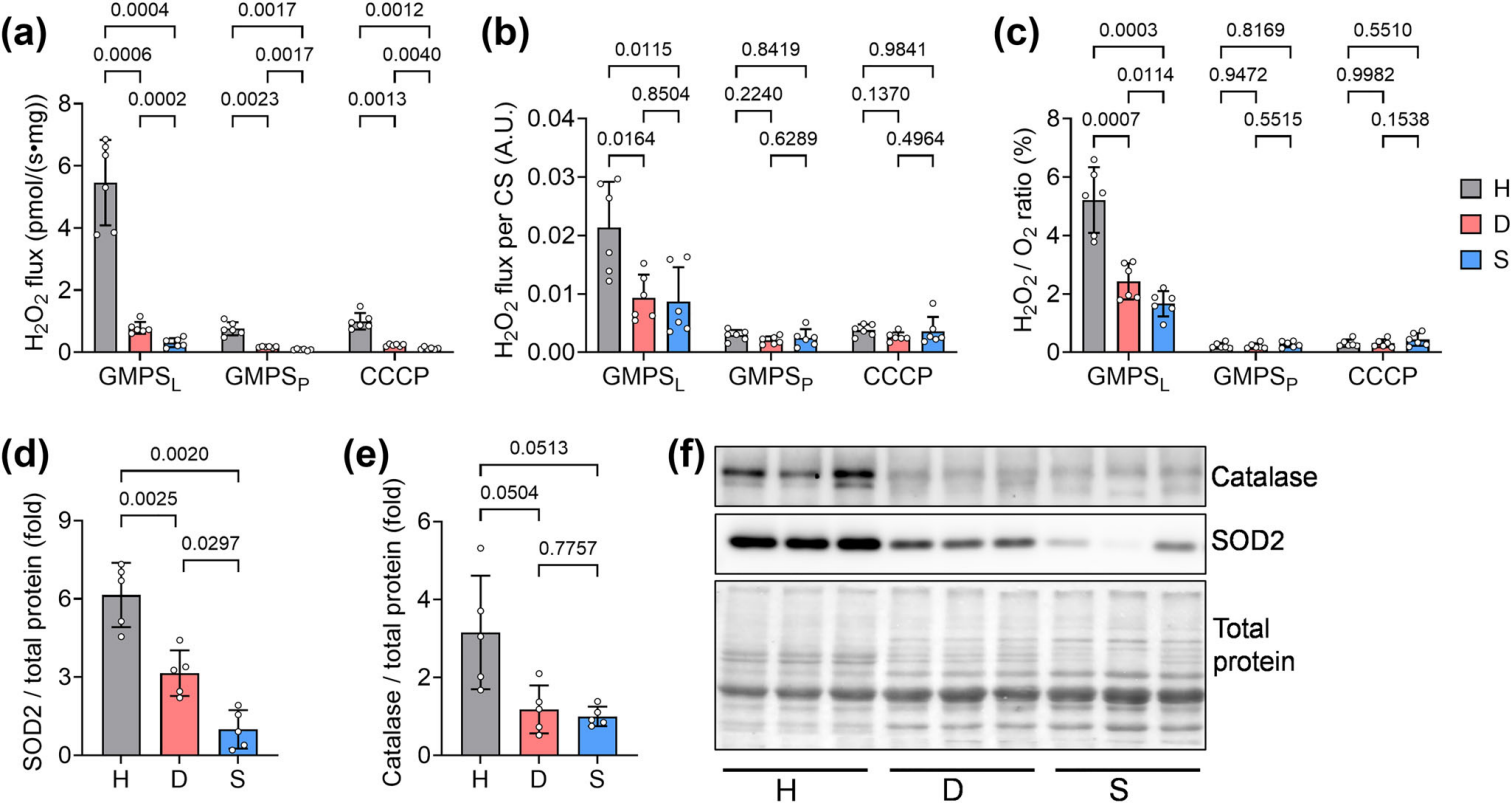
(a, b) Net rates of H_2_O_2_ emission at different respiratory states normalised to tissue wet mass (a) and citrate synthase activity (b). (c) H_2_O_2_ flux relative to O_2_ flux. (d, e) Protein expression levels of superoxide dismutase 2 (SOD2) (d) and catalase (e) relative to total protein stain and normalised to skeletal muscle samples for fold change. (f) Representative western blots for heart, diaphragm and skeletal muscles. D, diaphragm; H, heart; S, skeletal muscle. Two‐way ANOVA was used for (a, b, c), with values presented as means ± SD (*n* = 6). One‐way ANOVA was used for (d, e), with values presented as means ± SD (*n* = 5).

Expression levels of the antioxidant enzymes SOD2 and catalase were measured by immunoblotting. Heart tissues showed double the protein expression of SOD2 compared to diaphragm and ∼6‐fold that of skeletal muscle (*P* = 0.0025; Figure 2d). Heart mitochondria showed no significant difference in catalase expression compared to diaphragm and skeletal muscle (*P* = 0.051). The diaphragm showed greater SOD2 expression than skeletal muscle (*P* = 0.0297), with no difference in catalase expression.

## 4 DISCUSSION

This study concurrently compared mitochondrial respiration, H_2_O_2_ emission and key antioxidant enzymes across the heart, diaphragm and gracilis skeletal muscle. The initial hypothesis was that mitochondria in the heart had higher respiratory efficiency and stronger antioxidant systems than those in the other two muscle types. Our results demonstrated that per unit tissue mass, heart mitochondria had the highest O_2_ flux, mitochondrial respiratory efficiency, H_2_O_2_ flux, H_2_O_2_/O_2_ ratio and endogenous antioxidant levels. The differences in O_2_ flux per tissue mass between groups disappeared after normalising to CS activity, suggesting that mitochondrial abundance is the primary determinant of tissue‐level oxidative capacity. These findings are consistent with previous comparative investigations demonstrating that, although cardiac muscle exhibits higher absolute oxidative phosphorylation rates than skeletal muscle, normalisation to mitochondrial content markedly reduces inter‐tissue differences, suggesting a broadly conserved intrinsic respiratory capacity per mitochondrion across striated muscles (Park et al., 2014).

Skeletal muscles have a variety of anatomical forms and physiological functions throughout the body. The diaphragm displayed an intermediate phenotype between myocardium and limb skeletal muscle, aligning with its mixed voluntary and involuntary functional role and sustained rhythmic activity. However, mitochondrial preparation may alter the correlation between mitochondrial respiratory performance and muscle aerobic phenotype. Diaphragm mitochondria may exhibit lower intrinsic respiratory rates than those of limb skeletal muscle in isolated mitochondrial preparations (Garcia‐Cazarin et al., 2011). In contrast, studies using permeabilised fibres report that CS‐normalised differences in mitochondrial respiration between diaphragm and limb muscles are minimal (Hahn et al., 2019). Together with the present findings, these data suggest that mitochondrial density largely explains inter‐muscle differences in oxidative capacity under basal conditions. In contrast, intrinsic qualitative differences may become more apparent under different preparation methods, substrate conditions or physiological stress.

Net H_2_O_2_ emission was substantially greater during leak respiration than during ADP‐stimulated oxidative phosphorylation or uncoupled electron transfer states across all muscle types. This state dependence is well established, as non‐phosphorylating conditions elevate the membrane potential and increase the reduction of upstream electron carriers, thereby favouring superoxide formation at multiple sites within the electron transport system (Gnaiger, 2020; Murphy, 2009). Experimental models simulating rest versus exercise conditions similarly demonstrate higher mitochondrial ROS emission under resting, non‐phosphorylating conditions and markedly reduced emission during active oxidative phosphorylation (Goncalves et al., 2015). These findings reinforce the conclusion that mitochondrial ROS emission is strongly governed by the bioenergetic state rather than an invariant tissue property.

When normalised to tissue mass, cardiac muscle exhibited the highest H_2_O_2_ emission across respiratory states, consistent with its greater mitochondrial density. However, normalisation to CS activity substantially attenuated these differences, indicating comparable intrinsic ROS emission per mitochondrial unit during phosphorylating and uncoupled conditions. Notably, cardiac mitochondria displayed a higher ROS/O_2_ ratio during leak respiration, suggesting relatively greater electron leak under non‐phosphorylating conditions. Comparative analyses across muscle types have shown that, while OXPHOS capacity per mitochondrial content may be similar, coupling characteristics and non‐phosphorylating respiration can differ between tissues (Park et al., 2014). Elevated leak‐associated ROS/O_2_ in cardiac tissue may therefore reflect differences in membrane potential regulation, redox poise or electron transport system organisation.

Cardiac muscle demonstrated higher SOD2 expression, whereas catalase did not differ significantly between groups. Because net H_2_O_2_ emission reflects the balance between emission and scavenging, interpretation requires consideration of intramitochondrial detoxification systems. Measured extramitochondrial H_2_O_2_ represents the net outcome of production and consumption processes and may underestimate actual generation rates due to matrix scavenging systems (Makrecka‐Kuka et al., 2015). Muscles with high oxidative demand typically exhibit enhanced antioxidant capacity, including upregulation of SOD2 and catalase, particularly in response to sustained metabolic stress or training stimuli (Treberg et al., 2015). Because immunoblotting and respirometry were performed in separate cohorts, these group‐level findings cannot establish within‐animal coordination or a compensatory response.

Substrate selection and metabolic context further influence ROS emission profiles. Site‐specific superoxide generation has shown that the relative contribution of distinct electron transport system sites depends on substrate availability and redox state (Quinlan et al., 2012). Our protocol did not include fatty‐acid‐derived substrates, which may be relevant for cardiac muscle that relies on fatty acid oxidation in vivo, and may under‐represent pathway‐specific cardiac respiratory and redox behaviour under diverse fuel conditions in healthy and diseased states (Pham, 2026).

The intermediate mitochondrial phenotype in the diaphragm tissue has potential clinical relevance. Age‐related declines in diaphragm mitochondrial function (Torii et al., 1992) and chronic hypoxia (Khalilpour et al., 2025) have been shown to alter diaphragm mitochondrial content and distribution. Establishing baseline comparative bioenergetic and redox characteristics across striated muscles therefore provides a framework for interpreting disease‐associated remodelling.

Several strengths of the study enhance interpretability. Simultaneous high‐resolution respirometry and fluorometric detection allowed direct assessment of oxygen consumption and H_2_O_2_ emission under identical substrate–uncoupler–inhibitor conditions. Normalisation to both tissue mass and CS activity enabled differentiation between mitochondrial content‐driven and intrinsic functional differences. Limitations include the use of tissue homogenates rather than permeabilised fibres, which may disrupt mitochondrial membranes and differentially affect the dense cardiac mitochondrial network. Without a cytochrome *c* test, outer‐membrane integrity was not assessed directly. Citrate synthase activity is a biochemical proxy rather than a direct measure of mitochondrial volume or number, and the modest sample sizes and limited antioxidant panel constrain inference.

In summary, respiratory flux per citrate synthase activity was similar under the present conditions, whereas coupling‐related ratios and leak‐state H_2_O_2_/O_2_ revealed tissue‐specific qualitative differences. Tissue‐level differences should be interpreted as associations with citrate synthase‐estimated mitochondrial content, not direct measures of mitochondrial abundance. Cardiac H_2_O_2_/O_2_ and antioxidant‐enzyme findings were obtained in separate cohorts and do not establish coordinated redox buffering. The present findings apply specifically to the gracilis muscle and should not be generalised to all limb skeletal muscles. These findings provide a comparative framework for future investigations into muscle‐specific mitochondrial adaptations in ageing and disease.

## AUTHOR CONTRIBUTIONS

Toan Pham and Anthony Hickey designed the study. Alex Chan, Claudia Wen and Toan Pham performed the experiments. Toan Pham was responsible for statistical data analysis and preparing figures. Alex Chan, Jules Devaux and Toan Pham co‐wrote the manuscript. All authors contributed to the data interpretation. All authors have read and approved the final version of this manuscript and agree to be accountable for all aspects of the work in ensuring that questions related to the accuracy or integrity of any part of the work are appropriately investigated and resolved. All persons designated as authors qualify for authorship, and all those who qualify for authorship are listed.

## CONFLICT OF INTEREST

None declared.

## GENERATIVE AI STATEMENT

No generative AI tools were used in the preparation of this manuscript.

## Data Availability

The data that support the findings of this study will be made available from the corresponding author upon request.
